# Transcriptomic analysis on the formation of the viable putative non-culturable state of beer-spoilage *Lactobacillus acetotolerans*

**DOI:** 10.1038/srep36753

**Published:** 2016-11-07

**Authors:** Junyan Liu, Yang Deng, Brian M. Peters, Lin Li, Bing Li, Lequn Chen, Zhenbo Xu, Mark E. Shirtliff

**Affiliations:** 1 College of Food Science and Engineering, South China University of Technology, Guangzhou 510640, P.R. China; 2 Department of Clinical Pharmacy, College of Pharmacy, University of Tennessee Health Science Center, Memphis, TN 38163, USA; 3 Guangdong Province Key Laboratory for Green Processing of Natural Products and Product Safety, Guangzhou 510640, P.R. China; 4 Department of Microbial Pathogenesis, School of Dentistry, University of Maryland, Baltimore MD 21201, USA

## Abstract

Lactic acid bacteria (LAB) are the most common beer-spoilage bacteria regardless of beer type, and thus pose significant problems for the brewery industry. The aim of this study was to investigate the genetic mechanisms involved in the ability of the hard-to-culture beer-spoilage bacterium *Lactobacillus acetotolerans* to enter into the viable putative non-culturable (VPNC) state. A genome-wide transcriptional analysis of beer-spoilage *L. acetotolerans* strains BM-LA14526, BM-LA14527, and BM-LA14528 under normal, mid-term and VPNC states were performed using RNA-sequencing (RNA-seq) and further bioinformatics analyses. GO function, COG category, and KEGG pathway enrichment analysis were conducted to investigate functional and related metabolic pathways of the differentially expressed genes. Functional and pathway enrichment analysis indicated that heightened stress response and reduction in genes associated with transport, metabolic process, and enzyme activity might play important roles in the formation of the VPNC state. This is the first transcriptomic analysis on the formation of the VPNC state of beer spoilage *L. acetotolerans*.

As a popular beverage, beer has been recognized as safe due to its high microbiological stability. A large variety of microorganisms are incapable of survival in beer due to environmental stressors, including high ethanol concentration (0.5% to 10% w/w), low pH (3.8–4.7), high carbondioxide concentration (approximately 0.5% w/w), extremely low oxygen content (<0.1 ppm), hop bitter compounds (approximately 17–55 ppm of iso-α-acids) and low concentrations of nutritive substances, etc.^1^,^2^. However, despite these unfavorable conditions for microbial growth, a few species of bacteria (primarily Lactobacilli and Pediococci) remain viable in this medium and are designated as beer-spoilage microorganisms. A number of lactic acid bacteria (LAB) have been well documented as a major cause of acidity and turbidity in beer^3^. As an important beer-spoilage bacterium, *Lactobacillus acetotolerans* has been previously identified as hard-to-culture and frequently occurs in south China breweries^4^. *L. acetotolerans* possesses the ability to produce lactic acid, acetic acid and diacetyl as end products of carbohydrate fermentation. This high diacetyl content imparts undesirable qualities to beer, including a buttery taste and oily mouthfeel.

Routine culture media for detecting beer-spoilage bacteria, such as de Man Rogosa Sharpe (MRS) and Nachweismediumfür bierschädliche Bakterien (NBB) agar^5^, are unable to identify particular species of LAB which eventually lead to spoilage, profit loss, and food safety concerns^4^,^5^. Incapability of detection is considered to be partially mediated by the ability of beer-spoilage bacteria to enter the viable putative non-culturable (VPNC) state. Bacterial cells in the VPNC state also exhibit various phenotypes, including reduced size, decreased metabolic activity, and alterations in membrane composition and cell wall structure^6^. Recently, the formation and resuscitation of the VPNC state in *L. acetotolerans* had been demonstrated^7^,^8^. Approximately 70 reported non-sporulating bacterial species are capable of forming the VPNC state as a survival strategy against environmental stressors, including starvation, extreme temperature, elevated osmotic pressure, oxygen concentration, or exposure to visible light.

In recent years, gene expression and differential protein expression occurring during the VPNC state have been widely studied^9^,^10^,^11^. Typically, real-time PCR-based approaches were used to investigate known genes involved in response to stress conditions, while RNA transcription was employed to assess the gene expression in the VPNC state^12^,^13^,^14^ leading to numerous studies reporting the formation and resuscitation of the VPNC state^15^,^16^,^17^,^18^. However, most of these studies were descriptive, detailing the changes associated with the VPNC state with regard to the physiological and biochemical aspects^19^,^20^. However, few studies have investigated the molecular mechanism governing entry into and resuscitation from the VPNC state. It is noteworthy that the gene expression leading to VPNC entry differs between bacterial species, is incredibly complex, and far from being well understood. It has been established that VPNC *L. acetotolerans* cells maintain beer spoilage ability and thus pose a serious threat to the brewery industry^6^. Thus, a comprehensive understanding of the genetic mechanisms involved in the formation of VPNC state is a significant first step in designing strategies to eliminate VPNC *L. acetotolerans* strains in the brewery setting.

RNA sequencing (RNA-seq) technology, a powerful and cost-effective tool, offers an unbiased approach to assess the transcriptional profiles of a species under various conditions^21^. However, there has been a paucity of studies to investigate the molecular mechanisms involved in the formation of the VPNC state using high throughput RNA-Seq. Thus, the present study aimed to determine global transcriptomic changes during the VPNC state to identify putative mechanisms involved in the formation of the VPNC state of three beer-spoilage *L. acetotolerans* strains. Nine samples (three defined states of three *L. aceotolerans* strains) were selected for RNA-seq analysis to represent the formation process of the VPNC state. In comparison to normal cells, gene expression associated with survival in beer and beer spoilage varied significantly in the mid-term state (56 days cultivation under stress condition) and VPNC state (112 days cultivation under stress condition).

## Results

### Formation of the VPNC state

Cellular viability and culturability of *L. acetotolerans* strains were evaluated once a week, and the formation of VPNC state by *L. acetotolerans* was obtained and verified after 16 ± 0.8 subcultures (112 ± 5.6 days).

### Overview of the transcriptional analysis

**Figure 1:**
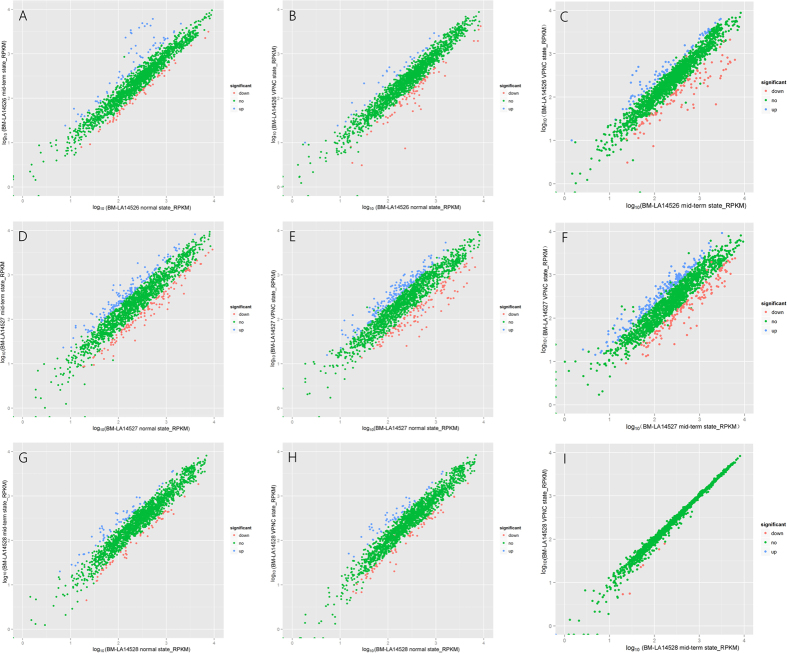
Differential expression level of BM-LA14526 normal state versus BM-LA14526 mid-term state (**A**), BM-LA14526 normal state versus BM-LA14526 VPNC state (**B**), BM-LA14526 mid-term state versus BM-LA14526 VPNC state (**C**), BM-LA14527 normal state versus BM-LA14527 mid-term state (**D**), BM-LA14527 normal state versus BM-LA14527 VPNC state (**E**), BM-LA14527 mid-term state versus BM-LA14527 VPNC state (**F**), BM-LA14528 normal state versus BM-LA14528 mid-term state (**G**), BM-LA14528 normal state versus BM-LA14528 VPNC state (**H**), and BM-LA14528 mid-term state versus BM-LA14528 VPNC state (**I**).

**Figure 2:**
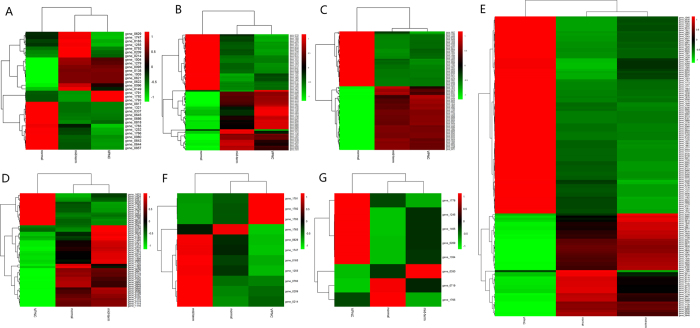
The expression level of common DEGs in normal state versus mid-term state and normal state versus VPNC state groups of *L. acetotolerans* strains BM-LA14526 (**A**), BM-LA14527 (**B**), BM-LA14528 (**C**), in mid-term state versus VPNC state and normal state versus VPNC state groups of *L. acetotolerans* strains BM-LA14526 (**D**), BM-LA14527 (**E**), and in normal, mid-term and VPNC state of *L. acetotolerans* strains BM-LA14526 (**F**), BM-LA14527 (**G**). The statistic graphs of expression level of common DEGs of *L. acetotolerans* strain BM-LA14528 in mid-term state versus VPNC state and normal state versus VPNC state groups, and in normal, mid-term and VPNC state are omitted considering single or no DEG in common.

cDNA libraries of nine *L. acetotolerans* samples were constructed, sequenced and generated with a total of 15,525,486–24,368,138 reads (Table 1). At least 89% of the total reads were mapped to the reference genome of *L. acetotolerans* strain BM-LA14527 (GenBank accession number: LTDX00000000). More than 43% of the total reads were mapped in proper pairs and the duplication rates range from 34% to 52%. Gene expression levels determined by the average values of Reads Per Kilobases per Million reads (RPKM) demonstrated that approximately 1800 total predicted genes were expressed in the normal, mid-term and VPNC states of the 3 *L. acetotolerans* strains (Table S1). Adjusted P value < 0.05 and │log_2_(fold change)│ >1 were used to identify DEGs. Diversity in identified DEGs was obtained, including 66/56 (normal state versus mid-term state for BM-LA14526), 35/74 (normal state versus VPNC state for BM-LA14526), 72/101 (mid-term state versus VPNC state for BM-LA14526), 141/120 (normal state versus mid-term state for BM-LA14527), 183/120 (normal state versus VPNC state for BM-LA14527), 206/142 (mid-term state versus VPNC state for BM-LA14527), 59/48 (normal state versus mid-term state for BM-LA14528), 51/58 (mid-term state versus VPNC state for BM-LA14528) and 0/7 (mid-term state versus VPNC state for BM-LA14528) up/down regulated DEGs (Fig. 1, Tables 2 and S2). Furthermore, 32, 77, and 82 common DEGs of *L. acetotolrans* strains BM-LA14526, BM-LA14527, and BM-LA14528 were identified in normal state versus mid-term state and normal state versus VPNC state groups (initial phase of VPNC state information), respectively (Fig. 2, Tables 2 and S3). As in mid-term state versus VPNC state and normal state versus VPNC state groups (latter phase of VPNC state information), 53, 143, and 1 common DEGs of the three *L. acetotolrans* strains were identified, respectively (Fig. 2, Tables 2 and S3). In addition, 11, 8, and 0 common DEGs were identified in normal state versus mid-term state, normal state versus VPNC state, and mid-term state versus VPNC state groups (whole process of VPNC state formation) of the three *L. acetotolrans* strains, respectively (Fig. 2, Tables 2 and S3). However, the three strains in the three comparative groups have no DEG in common. Typically, hop resistance gene *horA* (gene 1208) which associate with the ability to grow in beer was not DEG. The RPKM values of *horA* gene of three *L. acetotolerans* strains (BM-LA14526, BM-LA14527, BM-LA14528) in the VPNC state (390.379, 390.32, 380.111) were relatively lower than that in the mid-term state (424.962, 418.223, 373.506) and lower than that in normal state (555.858, 661.157, 604.654).

### GO functional analysis of DEGs

**Figure 3:**
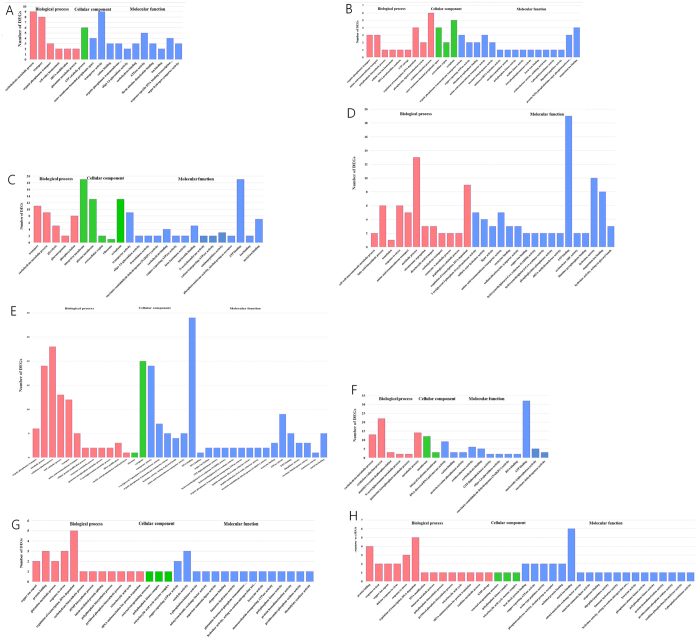
Significantly enriched GO terms of differentially expressed genes. The y-axis denotes the number of genes in a category. The x-axis represents the significantly enriched GO terms. (**A**) BM-LA14526 normal state versus BM-LA14526 mid-term state, (**B**) BM-LA14526 normal state versus BM-LA14526 VPNC state, (**C**) BM-LA14526 mid-term state versus BM-LA14526 VPNC state, (**D**) BM-LA14527 normal state versus BM-LA14527 mid-term state, (**E**) BM-LA14527 normal state versus BM-LA14527 VPNC state, (**F**) BM-LA14527 mid-term state versus BM-LA14527 VPNC state, (**G**) BM-LA14528 normal state versus BM-LA14528 mid-term state, (**H**) BM-LA14528 normal state versus BM-LA14528 VPNC state. The statistic graphs of BM-LA14528 mid-term state versus BM-LA14528 VPNC state group is omitted since no significantly enriched GO terms.

To understand the function of DEGs, GO function annotation and enrichment analysis were performed on the total, up-regulated, and down-regulated DEGs of three strains (Tables S4,S5,S6, with significantly enriched terms marked in bold). Significantly enriched GO terms composed of three specific categories (biological process, cellular component, and molecular function) of three strains were illustrated in Fig. 3.

As to the enriched GO terms in *L. acetotolrans* strain BM-LA14526, “organic phosphonate transport”, “outer membrane-bounded periplasmic space”, and “organic phosphonate transmembrane transporter activity” were significantly enriched in the categories of biological process (BP), cellular component (CC), and molecular function (MF), respectively. For the enrichment GO terms of up-regulated DEGs, “carbohydrate metabolic process” and “1-phosphofructokinase activity” were of significant enrichment in BP and MF categories, respectively. Of the 2 significantly enriched GO terms of down-regulated DEGs, “organic phosphonate transport” and “organic phosphonate transmembrane transporter activity” were identified in BP and MF categories, respectively.

In *L. acetotolerans* strain BM-LA14527, 3 GO terms in BP category (“metabolic process”, “amino acid transmembrane transport”, “coenzyme A metabolic process”) and 6 GO terms in MF category (“1-acylglycerol-3-phosphate O-acyltransferase activity”, “amino acid transmembrane transporter activity”, “hydrolase activity, acting on glycosyl bonds”, “copper ion binding”, “hydroxymethylglutaryl-CoA reductase (NADPH) activity”, “hydroxymethylglutaryl-CoA synthase activity”) were significantly enriched. As to significantly enriched GO terms of up-regulated DEGs, “metabolic process” and “coenzyme A metabolic process” were identified in BP category, and “coenzyme binding”, “hydroxymethylglutaryl-CoA reductase (NADPH) activity” and “hydroxymethylglutaryl-CoA synthase activity” were identified in the category of MF. Moreover, a total of 4 GO terms (“transport” and “amino acid transmembrane transport” in BP category, “1-acylglycerol-3-phosphate O-acyltransferase activity” and “amino acid transmembrane transporter activity” in MF category) were determined to be significantly enriched terms of the down-regulated DEGs.

The *L. acetotolrans* strain BM-LA14528 had the most significantly enriched GO terms in common. Among the 8 significantly enriched terms in BP category, “regulation of transcription, DNA-dependent”, “response to stress”, “protein folding”, and “copper ion export” were the dominant terms. Three significantly enriched terms in CC category were “external encapsulating structure”, “polyphosphate kinase complex”, and “tricarboxylic acid cycle enzyme complex”. The GO term “copper-exporting ATPase activity” highly represented the 11 terms of significant enrichment in MF category. Of the 17 significantly enriched GO terms of the up-regulated DEGs, the dominant terms in BP and MF categories were the same with that of the total DEGs. As for the significantly enriched GO terms of the down-regulated DEGs, 4, 3, and 4 terms were identified in the categories of BP, CC, and MF, respectively, and the dominant term in CC category was the same with that of the total DEGs.

### COG functional category analysis of DEGs

**Figure 4:**
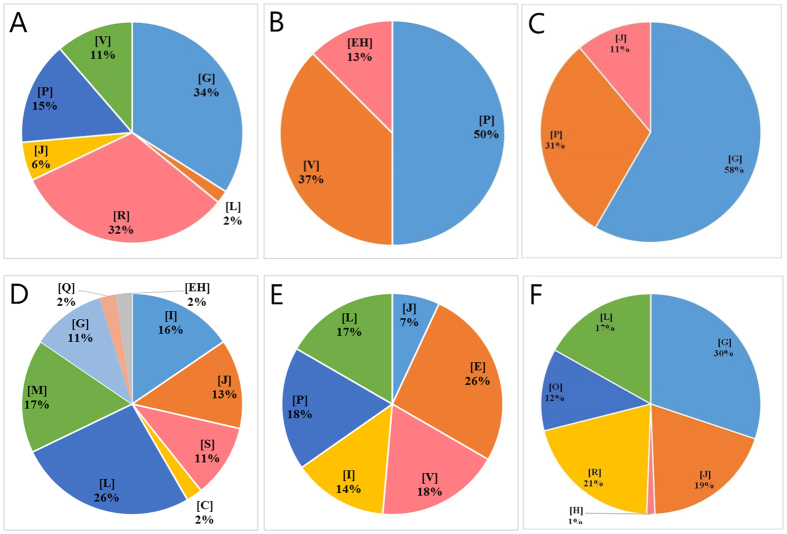
Significantly enriched COG terms of differentially expressed genes. (**A**) BM-LA14526 normal state versus BM-LA14526 mid-term state, (**B**) BM-LA14526 normal state versus BM-LA14526 VPNC state, (**C**) BM-LA14526 mid-term state versus BM-LA14526 VPNC state, (**D**) BM-LA14527 normal state versus BM-LA14527 mid-term state, (**E**) BM-LA14527 normal state versus BM-LA14527 VPNC state. (**F**) BM-LA14527 mid-term state versus BM-LA14527 VPNC state, The statistic graphs of the three groups of BM-LA14528 are omitted due to the single significantly enriched COG terms. [V] Defense mechanisms, [G] Carbohydrate transport and metabolism, [P] Inorganic ion transport and metabolism, [J] Translation, ribosomal structure and biogenesis, [R] General function prediction only, [L] Replication, recombination and repair, [EH] Thiamine pyrophosphate-requiring enzymes, [Q] Secondary metabolites biosynthesis, transport and catabolism, [S] Function unknown, [M] Cell wall/membrane/envelope biogenesis, [C] Energy production and conversion, [I] Lipid transport and metabolism, [E] Amino acid transport and metabolism, [O] Posttranslational modification, protein turnover, chaperones, [H] Coenzyme transport and metabolism.

To further understand the putative function of DEGs, COG annotation and enrichment analysis were performed on the total, up-regulated, and down-regulated DEGs of 6 groups (Tables S7,S8,S9, with significantly enriched terms marked in bold) and significantly enriched COG categories of the three strains were also analyzed (Fig. 4).

For the COG categories acquired by *L. acetotolerans* strain BM-LA14526, “[P] Inorganic ion transport and metabolism” (also significantly enriched in the COG categories of down-regulated DEGs) and “[V] Defense mechanisms” were significantly enriched. However, no COG category of the up-regulated DEGs was found to be of significant enrichment. Among the three strains, *L. acetotolerans* strain BM-LA14527 had the most significantly enriched COG categories, with 3 (“[L] Replication, recombination and repair”, “[I] Lipid transport and metabolism”, and “[J] Translation, ribosomal structure and biogenesis”), 1 (“[I] Lipid transport and metabolism”) and 1 (“[E] Amino acid transport and metabolism”) of the total, up-regulated and down-regulated DEGs, respectively. In the COG categories enriched by *L. acetotolrans* strain BM-LA14528, only two categories (“[O] Posttranslational modification, protein turnover, chaperones” and “[KT] COG1983 Putative stress-responsive transcriptional regulator”) of the up-regulated DEGs were determined significantly enriched.

### KEGG pathway analysis of DEGs

**Figure 5:**
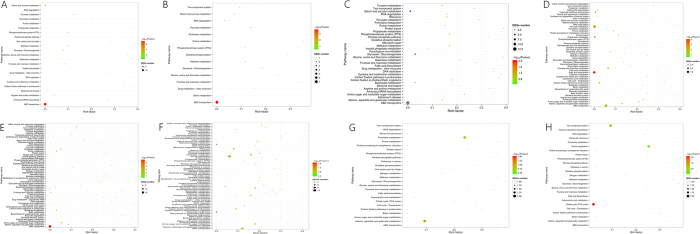
KEGG pathway enrichment of differentially expressed genes. (**A**) BM-LA14526 normal state versus BM-LA14526 mid-term state, (**B**) BM-LA14526 normal state versus BM-LA14526 VPNC state, (**C**) BM-LA14526 mid-term state versus BM-LA14526 VPNC state, (**D**) BM-LA14527 normal state versus BM-LA14527 mid-term state, (**E**) BM-LA14527 normal state versus BM-LA14527 VPNC state, (**F**) BM-LA14527 mid-term state versus BM-LA14527 VPNC state, (**G**) BM-LA14528 normal state versus BM-LA14528 mid-term state, (**H**) BM-LA14528 normal state versus BM-LA14528 VPNC state. The statistic graphs of BM-LA14528 mid-term state versus BM-LA14528 VPNC state group is omitted due to the single KEGG pathway.

To identify the pathways of DEGs, DEGs were mapped to the KEGG database and KEGG enrichment analysis were performed on the total, up-regulated, and down-regulated DEGs of three strains (Fig. 5, Tables S10,S11,S12, with significantly enriched terms marked in bold).

Of total and up-regulated DEGs in *L. acetotolerans* strain BM-LA14526, “ABC transporters” was significantly enriched. However, no significantly enriched pathways were in common with the down-regulated DEGs. As for *L. acetotolerans* strain BM-LA14527, 2 (“Fatty acid biosynthesis” and “Synthesis and degradation of ketone bodies”), 4 (“Fructose and mannose metabolism”, “Synthesis and degradation of ketone bodies”, “Valine, leucine and isoleucine degradation”, and “Glycerolipid metabolism”), and 1 (“ABC transporters”) were significantly enriched in the total, up-regulated, and down-regulated DEGs, respectively. The pathways of “Biotin metabolism”, “Pathways in cancer”, “Protein processing in endoplasmic reticulum”, and “Renal cell carcinoma” were identified to be significant enrichment in *L. acetotolerans* strain BM-LA14528. In addition, “Biotin metabolism” and “Renal cell carcinoma” were also significantly enriched pathways of down-regulated DEGs, while “Protein processing in endoplasmic reticulum” was also significantly enriched in pathways of up-regulated DEGs.

### qRT-PCR validation

Ten genes with different expression profiles were selected to validate the results of RNA-seq. The representative 10 genes were selected based on three criteria: (i) Gene function. (ii) Expression level. (iii) Gene position in the genome. The 10 genes (5 up-regulated and 5 down-regulated) were selected from different significantly enriched GO terms, COG categories and KEGG pathways, and located in different positions in the genome. According to the results, the mRNA levels of the 10 representative genes determined by qRT-PCR were consistent with those from the RNA-Seq analysis.

## Discussion

In nature, bacteria are unavoidably exposed to variety of stress conditions, thus strategies have evolved to enhance survival under a broad array of conditions. Despite the prevalence of the VPNC state as one of the survival strategies adopted by non-sporulating bacteria, few genetic mechanisms involved in the VPNC state have been elucidated. Thorough understanding of the global gene expression profiling of the VPNC state may provide valuable insights into genetic mechanisms of bacterial survival under stress conditions and the VPNC state itself. To our knowledge, studies on genome-wide expression profiling of the mid-term and VPNC state in food spoilage bacteria are currently unavailable.

Our previous study demonstrated that *L. acetotolerans* strains are hard to culture in routine medium, capable of entering into and resuscitating from the VPNC state, and maintain beer spoilage capacity during VPNC conditions^6^. In this study, RNA-seq and bioinformatics analysis were performed to map global gene expression associated with VPNC *L. acetotolerans* strains, with the goal of identifying the genetic mechanisms contributing to the VPNC state. After sequencing and mapping to the reference genome, gene expression levels were measured by RPKM and DEGs identified by the adjusted P value < 0.05 and │log_2_(fold change)│ >1. According to the expression variation in different phase, DEGs identified in the three *L. acetotolerans* strains were divided into 3 types, designated type I (the initial phase, from normal to mid-term state), II (the latter phase, from mid-term to VPNC state), and III (the whole process of the VPNC formation) (Table 2). Among the type I DEGs, 9/12, 34/35, and 43/39 were up/down regulated in *L. acetotolerans* strains BM-LA14526, BM-LA14527, and BM-LA14528, respectively. Also, 12/30, 89/46, and 0/7 DEGs were identified to be up/down regulated among type II DEGs. As for the 11 type III DEGs of strain BM-LA14526, 7 were up-regulated in the initial phase and down-regulated in the latter phase, 3 were down-regulated in the initial phase and up-regulated in the latter phase, and 1 was down-regulated in the whole process. For type III DEG of strain BM-LA14527, 4/1 were up/down regulated in the whole process, 1 was up-regulated in the initial phase and down-regulated in the latter phase, and 2 were down-regulated in the initial phase and up-regulated in the latter phase. As none of common DEG was found in all of the three strains, the genetic mechanisms of the VPNC entry might vary widely from strain to strain. Notably in strain BM-LA14528, 82 type I DEGs were identified, with only one type II DEG and no type III DEG obtained, which suggested the initial phase of VPNC state formation may play more important role than the latter phase during the complex process of VPNC state formation. Since the *horA* gene expression levels were diminishing from normal state via mid-term state to the VPNC state, the growth ability of the three *L. acetotolerans* strains in beer might decrease during the formation of the VPNC state. However, the *horA* gene was not found in DEGs from all comparison groups, indicating insignificant effect of VPNC state formation on the growth of *L. acetotolerans* strains in beer.

The functions and pathways of DEGs associated with the formation of the three VPNC *L. acetotolerans* strains were compared by enrichment analysis. Analysis of GO functional enrichment demonstrated that significantly enriched terms of up-regulated DEGs in *L. acetotolerans* strains were related to “carbohydrate metabolic process”, “pyruvate metabolism”, “1-phosphofructokinase activity”, “metabolic process”, “coenzyme A metabolic process”, “hydroxymethylglutaryl-CoA reductase (NADPH) activity”, “hydroxymethylglutaryl-CoA synthase activity”, “coenzyme binding”, “regulation of transcription, DNA-dependent”, “response to stress”, “protein folding”, “copper ion export”, and “copper-exporting ATPase activity”. It was reported that lactobacilli modify the transport and metabolism of carbohydrates to adapt to the carbon source available in different media and to withstand a panel of stresses^22^,^23^,^24^,^25^. During the formation of the VPNC state, *L. acetotolerans* strains may up-regulate the carbohydrate metabolic process to withstand stress or adapt to depletion of available nutrients in the culture medium. Activity of NADPH-generating systems was proposed to be critical for maintenance of the viability of VPNC cells^26^. Su *et al*. induced the VPNC state of *Rhodococcus* and analyzed the transcriptome of VPNC state determining that “coenzyme binding” was the dominant up-regulated GO term in the molecular function category^27^,^28^. Coupled with our results, these findings indicate that “coenzyme binding” may somehow play a role in the formation of the VPNC state in more than one species. Environmental stress conditions affect the abundance of proteins involved in transcriptional regulation^29^. Regulation of bacterial signaling and gene transcription is often mediated by two-component regulatory systems, consisting of a membrane-bound histidine protein kinase and a cytoplasmic response regulator^30^. Several up-regulated genes in the category of “stress resistance”, “regulation of transcription, DNA-dependent” and “response to stress” potentially indicate involvement of their protein products in the VPNC state^26^.

In addition, the function of down-regulated DEGs involving the GO categories of “organic phosphonate transport”, “organic phosphonate transmembrane transporter activity”, “amino acid transmembrane transport”, “transport”, “1-acylglycerol-3-phosphate O-acyltransferase activity”, “amino acid transmembrane transporter activity”, “polyphosphate biosynthetic process”, “tricarboxylic acid cycle”, “tRNA aminoacylation for protein translation”, “L-asparagine biosynthetic process”, “external encapsulating structure”, “polyphosphate kinase complex”, “tricarboxylic acid cycle enzyme complex”, “aspartate-ammonia ligase activity”, “fumarate hydrolase activity”, “hydrolase activity, acting on carbon-nitrogen (but not peptide) bonds, in linear amides”, and “polyphosphate kinase activity”. Similar to previously observed changes in morphology^7^, the reduction of transport process, transporter activity, biosynthetic process, translation, and enzyme activity could be survival strategies allowing VPNC cells to minimize biochemical activity or nutrient-dependence to adapt to environmental stress conditions.

As for COG category enrichment analysis, significantly enriched up-regulated categories were associated with “[V] Defense mechanisms”, “[I] Lipid transport and metabolism”, “[O] Posttranslational modification, protein turnover, chaperones”, and “[KT] COG1983 Putative stress-responsive transcriptional regulator”. “[P] Inorganic ion transport and metabolism” and “[E] Amino acid transport and metabolism” were significantly enriched down-regulated categories. The control of amino acid transport and metabolism might be necessary for maintaining the carbon-nitrogen balance in VPNC cells^26^, with amino acid metabolism-related genes regulated in response to environmental signals, such as an abundant nutrient supply or nutrient deprivation^30^. The up-regulated categories enriched in “defense mechanisms” and “stress-responsive transcriptional regulator” suggests that *L. acetotolerans* engages defense and stress response systems to finally enter into the VPNC state in order to survive under stress conditions, including brewed beverages. On the other hand, reducing amino acid transport and metabolism may be a strategy to decrease energy requirements and enhance the function of survival systems.

The enrichment analysis of KEGG pathway indicated that, “ABC transporters”, “Pyruvate metabolism”, “Synthesis and degradation of ketone bodies”, “Valine, leucine and isoleucine degradation”, “Fructose and mannose metabolism”, “Protein processing in endoplasmic reticulum” were dominant up-regulated pathways. The “ABC transporters” pathway is important for the import of essential nutrients and export of toxic molecules in bacteria^31^. These transporters may provide prerequisite nutrients and metabolites for VPNC cell formation or continually rid cells of toxins in adverse envrionments^26^. For lactobacilli, modification of pyruvate metabolism via enzymatic increase had been verified for bacterial survival under acid stress or starvation conditions^32^,^33^,^34^,^35^,^36^,^37^. In the current study, the up-regulation of DEGs involved in “pyruvate metabolism” pathway and GO term suggested that *L. acetotolerans* up-regulated related enzymes to survive in beer (acid and starved environment). The two up-regulated DEGs designated gene 0267 and gene 0269, which were both involved in “Synthesis and degradation of ketone bodies” and “Valine, leucine and isoleucine degradation” pathways of *L. acetotolerans* strain BM-LA14527, encoded hydroxymethylglutaryl-CoA synthase and acetyl-CoA acetyltransferase, respectively. It was reported that *L. buchneri* increased the amount of enzymes related to the metabolism of arginine, proline, glycine, serine, and threonine under ethanol stress conditions^38^, while *L. brevis* increased the amount of cysteine sulfinate desulfinase/cysteine desulfurase-related enzyme and pyridoxal phosphate-dependent decarboxylase under hop-stressed conditions^39^. Similar to *L. buchneri* and *L. brevis, L. acetotolerans* might increase these genes in response to alcohol or hops. Formation of the VPNC state was also affected by other up-regulated genes with unknown functions. A total of 5 up-regulated DEGs associated with the “Fructose and mannose metabolism” pathway in *L. acetotolerans* strain BM-LA14527 encoded fructokinase, tagatose-6-phosphate kinase, PTS fructose transporter subunit IIC, mannitol-1-phosphate 5-dehydrogenase, and glycosyl transferase. The single up-regulated DEG (gene 1112) involved in “Protein processing in endoplasmic reticulum” pathway *L. acetotolerans* strain BM-LA14528 encoded molecular chaperone.

Dominant down-regulated pathways in *L. acetotolerans* strains included “Fatty acid biosynthesis”, “Biotin metabolism”, “Cyanoamino acid metabolism”. The presence of such compounds increase stress on lactobacilli causing decreased synthesis of enzymes involved in fatty acid biosynthesis^36^,^40^,^41^,^42^,^43^,^44^. However, increases in fatty acid biosynthetic enzymes in lactobacilli have been previously shown to protect the cellular membrane against environmental stresses^45^. The down-regulation of genes involved in “Fatty acid biosynthesis” pathway in the three *L. acetotolerans* strains might be a stress response and survival part of the genetic mechanism in the formation of the VPNC state. Furthermore, two other down-regulated genes encoded biotin-(acetyl-CoA-carboxylase) ligase (involved in “Biotin metabolism” pathway) and asparagine synthase (involved in “Cyanoamino acid metabolism” pathway), might somehow affect the formation of VPNC *L. acetotolerans* strain BM-LA14528.

It is noteworthy that the significantly enriched GO terms, COG categories, and KEGG pathways identified in the VPNC state between the three *L. acetotlerans* used in this study differed considerably from one another and findings that were previously published using other bacterial species^26^,^27^,^38^. Thus, the genetic mechanisms contributing to VPNC entry and maintenance are still loosely defined and will require validation by gene knockout studies to confirm their role in this biological process.

This study clarified the genome-wide transcriptional variation in the formation of the VPNC *L. acetotolrans* strains. Gene expression of the VPNC state indicated strong involvement of stress response pathways and reductions in key transport, metabolic, and enzymatic processes may play an important role in its formation. Moreover, these results determined that the molecular response underlying VPNC entry is complicated and differs between strains and species. It is unclear whether these responses are conserved in all beer types or all conditions that promote VPNC formation but are important questions for further investigation. Additionally, further studies should be conducted to determine whether other beer-spoilage bacteria can enter into the VPNC state and if they express the similar transcriptomic changes as *L. acetotolerans*. Elucidating how beer-spoilage bacteria regulate VPNC entry will afford opportunities to inhibit this process and alleviate beverage contamination and food safety concerns in the brewing industry.

## Methods

### Bacterial strains and growth conditions

Three *L. acetotolerans* strains, BM-LA14526, BM-LA14527, and BM-LA14528, were isolated from turbid finished beers (identified to be spoilage) in Guangzhou (China) in 2014 and grown anaerobically at 26 °C in MRS broth (Oxoid, UK).

### Induction of the VPNC state

Induction of the VPNC state in strains BM-LA14526, BM-LA14527, and BM-LA14528 was conducted by beer subculture^7^. Firstly, the assay of prolonged adaptation to beer described by Suzuki *et al*. was carried out^8^. Since *L. acetotolerans* strain was found to be unable to grow in high-bitterness beers, Chinese beers were used for induction of the VPNC state. Approximately 10^7^ cells of *L. acetotolerans* BM-LA14526, BM-LA14527, and BM-LA14528 were inoculated and anaerobically subcultured at 26 °C in 10 mL aliquots of the degassed commercial beer. The interval of each subculture was 7 days. Afterwards, the bacterial strain was induced to entry into VPNC state by continuous passage in beers. 1 mL exponentially growing bacterial strain which was considered to be the 0th generation was inoculated to 10 mL degassed beer and anaerobically cultured at 26 °C for 7 days to be the 1st generation. Then 1 mL 1st generation culture was inoculated to 10 mL degassed beer and anaerobically cultured at 26 °C for 7 days to be the 2^nd^ generation, the amount of 2^nd^ generation culture were measured by plate colony counting method. All the studies related to entry into VPNC state were performed in independent triplicate biological experiments. The number of culturable cells, total cells, and cell viability were accessed by a conventional plate culture procedure, AODC method, and Live/Dead BacLight bacterial viability kit (Molecular Probes, USA), respectively^7^.

### Preparation of RNA-seq samples

The samples in normal, mid-term, and VPNC state were prepared by 0, 56, and 112 days cultivation under stress condition, respectively. Samples (n = 9; BM-LA14526 normal state, BM-LA14526 mid-term state, BM-LA14526 VPNC state, BM-LA14527 normal state, BM-LA14527 mid-term state, BM-LA14527 VPNC state, BM-LA14528 normal state, BM-LA14528 mid-term state, BM-LA14528 VPNC state) were cultured as described and prepared for RNA-seq.

### RNA extraction, library construction and sequencing

Total RNA of *L. acetotolerans* samples were extracted using the Bacterial Total RNA Extraction kit (Sigma-Aldrich, USA) according to the manufacturer’s instruction. The quality of total RNA was determined using Agilent 2100 Bioanalyzer (Agilent Technologies, Santa Clara, CA). RNase-free DNase I (Ambion Inc., Austin, TX, USA) and MICROBExpress^TM^ kit (Ambion Inc., Austin, TX, USA) were used to remove trace DNA and ribosomal RNAs, respectively. The mRNA was fragmented ultrasonically and then reverse transcribed to cDNA. RNA-seq libraries were constructed using the Illumina Paired End Sample Prep kit and all samples were sequenced using the Illumina Hiseq 2500 platform.

### RNA-seq data analysis

Raw reads were generated from image data and stored in FASTQ format. All the raw data have been deposited in the NCBI Short Read Archive (SRA) database under accession no. SRX1687039. Clean reads were aligned to *L. acetotolerans* BM-LA14527 (GenBank accession no. LTDX00000000) using TopHat^46^. Only mismatches and read gaps with no more than 2 bases were allowed in the alignment. Gene coverage was calculated by the percentage of genes covered by reads. The nine samples were combined to 9 comparison groups (BM-LA14526 normal state versus BM-LA14526 mid-term state, BM-LA14526 normal state versus BM-LA14526 VPNC state, BM-LA14526 mid-term state versus BM-LA14526 VPNC state, BM-LA14527 normal state versus BM-LA14527 mid-term state, BM-LA14527 normal state versus BM-LA14527 VPNC state, BM-LA14527 mid-term state versus BM-LA14527 VPNC state, BM-LA14528 normal state versus BM-LA14528 mid-term state, BM-LA14528 normal state versus BM-LA14528 VPNC state, and BM-LA14528 mid-term state versus BM-LA14528 VPNC state) to determine differentially expressed genes (DEGs) under each culture condition. DEGs were identified using DEGseq panormalage^47^ and the method described by Audic S.^48^. The gene expression level was measured using RPKM method^49^ and P values adjusted using the edgeR panormalage^50^. Genes with an adjusted P value < 0.05 and │log_2_(fold change)│ >1 were identified as DEGs. Gene Ontology (GO)^51^, Clusters of Orthologous Groups (COG)^52^ and Kyoto Encyclopedia of Genes and Genomes (KEGG) pathway^53^ analysis were performed to determine the putative function of DEGs.

### Quantitative real-time RT-PCR (qRT-PCR) validation

In order to validate the RNA-seq data, qRT-PCR was performed to quantify the mRNA transcripts of 10 selected DEGs using the CFX96 qRT-PCR System (Bio-Rad, USA) according to the manufacturer’s instructions. The RNA samples were extracted using Bacterial Total RNA Extraction kit (Sigma-Aldrich, USA) and treated with RNase-free DNase I to remove DNA contamination. Each qRT-PCR reaction was conducted in a final volume of 25 μL. The thermal cycling profile was as follows: 42 °C for 60 min and 72 °C for 10 min; 45 cycles of 95 °C for 10 s, 60 °C for 30 s, 70 °C for 1 min and a final extension of 68 °C for 7 min. Negative control samples containing sterile water were also included. The cycle threshold values (C_T_) were determined and the relative fold differences were calculated by the 2^−ΔΔC^_T_ method^54^ using *16S rRNA* as the reference gene. Three independent experiments were run in triplicate.

### Statistical analysis

Data are presented as mean ± standard deviation (SD) of three independent biological replicates. Statistical comparisons were made by one-way analysis of variance (ANOVA) followed by Hypergeometric test (R language). A p-value < 0.05 was considered to be statistically significant.

## Additional Information

**How to cite this article**: Liu, J. *et al*. Transcriptomic analysis on the formation of the viable putative non-culturable state of beer-spoilage *Lactobacillus acetotolerans. Sci. Rep.* **6**, 36753; doi: 10.1038/srep36753 (2016).

**Publisher’s note**: Springer Nature remains neutral with regard to jurisdictional claims in published maps and institutional affiliations.

## Supplementary Material

Supplementary Table S1

Supplementary Table S2

Supplementary Table S3

Supplementary Table S4

Supplementary Table S5

Supplementary Table S6

Supplementary Table S7

Supplementary Table S8

Supplementary Table S9

Supplementary Table S10

Supplementary Table S11

Supplementary Table S12

## Figures and Tables

**Table 1:** Overview of the RNA-Seq statistics.

Sample name	BM-LA14526 normal	BM-LA14526 mid-term	BM-LA14526 VPNC	BM-LA14527 normal	BM-LA14527 mid-term	BM-LA14527 VPNC	BM-LA14528 normal	BM-LA14528 mid-term	BM-LA14528 VPNC
Total reads	21,600,338 (100%)	20,654,358 (100%)	15,525,486 (100%)	24,368,138 (100%)	17,457,014 (100%)	18,939,482 (100%)	22,294,928 (100%)	18,457,718 (100%)	17,205,716 (100%)
Total mapped	20,470,722 (94.77%)	19,569,997 (94.75%)	14,251,928 (91.80%)	23,216,282 (95.27%)	15,620,186 (89.48%)	18,223,708 (96.22%)	21,186,600 (95.03%)	16,523,467 (89.52%)	15,486,900 (90.01%)
Multiple mapped	36,045 (0.17%)	20,995 (0.10%)	74,471 (0.48%)	29,973 (0.12%)	22,786 (0.13%)	13,647 (0.07%)	27,634 (0.12%)	25,583 (0.14%)	1,961 (0.01%)
Uniquely mapped	20,434,677 (94.60%)	19,549,002 (94.65%)	14,177,457 (91.32%)	23,186,309 (95.15%)	15,597,400 (89.35%)	18,210,061 (96.15%)	21,158,966 (94.90%)	16,497,884 (89.38%)	15,484,939 (90.00%)
Read 1 mapped	10,235,304 (47.38%)	9,783,949 (47.37%)	7,125,546 (45.90%)	11,607,869 (47.64%)	7,809,982 (44.74%)	9,111,555 (48.11%)	10,592,610 (47.51%)	8,261,955 (44.76%)	7,743,832 (45.01%)
Read 2 mapped	10,235,418 (47.39%)	9,786,048 (47.38%)	7,126,382 (45.90%)	11,608,413 (47.64%)	7,810,204 (44.74%)	9,112,153 (48.11%)	10,593,990 (47.52%)	8,261,512 (44.76%)	7,743,068 (45.00%)
Reads mapped to plus strand	10,263,988 (47.52%)	9,805,062 (47.47%)	7,149,891 (46.05%)	11,622,821 (47.70%)	7,831,148 (44.86%)	9,126,824 (48.19%)	10,605,945 (47.57%)	8,286,177 (44.89%)	7,765,000 (45.13%)
Reads mapped to minus trand	10,206,734 (47.25%)	9,764,935 (47.28%)	7,102,037 (45.74%)	11,593,461 (47.58%)	7,789,038 (44.62%)	9,096,884 (48.03%)	10,580,655 (47.46%)	8,237,290 (44.63%)	7,721,900 (44.88%)
Reads mapped in proper pairs	10,088,758 (46.71%)	9,611,492 (46.53%)	6,974,625 (44.92%)	11,341,306 (46.54%)	7,603,573 (43.56%)	8,958,329 (47.30%)	10,374,070 (46.53%)	8,051,562 (43.62%)	7,554,130 (43.90%)
Duplication	10,919,783 (50.55%)	10,650,511 (51.57%)	6,285,061 (40.48%)	12,336,153 (50.62%)	7,652,263 (43.83%)	9,506,626 (50.19%)	10,441,104 (46.83%)	6,359,654 (34.46%)	5,899,639 (34.29%)
Expressed genes	1814	1818	1815	1818	1812	1819	1819	1819	1814

**Table 2:** Statistics of DEGs.

DEGs	Normal state versus mid-term state	Normal state versus VPNC state	Mid-term state versus VPNC state	Initial phase	Latter phase	Whole process	Type I	Type II	Type III
BM-LA14526	122	109	173	32	53	11	21	42	11
BM-LA14527	261	303	348	77	143	8	69	135	8
BM-LA14528	107	109	7	82	1	0	82	1	0

DEGs in the initial phase: common DEGs in normal state versus mid-term state and normal state versus VPNC state groups; DEGs in the latter phase: common DEGs in mid-term state versus VPNC state and normal state versus VPNC state groups; DEGs in the whole process: DEGs both in the initial and latter phase.
